# Promoting Re-Epithelialization in Diabetic Foot Wounds Using Integrative Therapeutic Approaches

**DOI:** 10.3390/bioengineering12101053

**Published:** 2025-09-29

**Authors:** Lucia Bubulac, Iuliana-Raluca Gheorghe, Elisabeth Ungureanu, Claudia Florina Bogdan-Andreescu, Cristina-Crenguța Albu, Consuela-Mădălina Gheorghe, Ovidiu Mușat, Irina Anca Eremia, Cristina Aura Panea, Alexandru Burcea

**Affiliations:** 1 Department of Family Medicine, Faculty of Medicine, “Carol Davila” University of Medicine and Pharmacy, 020021 Bucharest, Romania; lucia.bubulac@umfcd.ro (L.B.); irina.eremia@umfcd.ro (I.A.E.); 2 Department of Marketing and Medical Technology, Faculty of Medicine, “Carol Davila” University of Medicine and Pharmacy, 020021 Bucharest, Romania; raluca.gheorghe@umfcd.ro; 3 Independent Researcher, 020021 Bucharest, Romania; elisabeth.ungureanu@gmail.com; 4 Department of Speciality Disciplines, “Titu Maiorescu” University, 031593 Bucharest, Romania; claudia.andreescu@prof.utm.ro (C.F.B.-A.); alexandru.burcea@prof.utm.ro (A.B.); 5 Department of Genetics, Faculty of Dentistry, “Carol Davila” University of Medicine and Pharmacy, 020021 Bucharest, Romania; 6 Department of Ophthalmology, Faculty of Medicine, “Carol Davila” University of Medicine and Pharmacy, 020021 Bucharest, Romania; ovidiu.musat@umfcd.ro; 7 Department of Neuroscience, Faculty of Medicine, “Carol Davila” University of Medicine and Pharmacy, 020021 Bucharest, Romania; cristina.panea@umfcd.ro

**Keywords:** diabetic foot wounds, re-epithelialization, ozone therapy, gut microbiome, low-frequency electromagnetic therapy, integrative medicine, chronic wounds, oxidative stress, wound healing

## Abstract

**Background**: Diabetes mellitus is a heterogeneous chronic disease with an increasing global prevalence. In Romania, 11.6% of the population is affected, yet only 6.46% receive treatment. Among diabetic patients, 15–25% develop skin lesions that may progress to ulceration and necrosis, significantly impairing quality of life and increasing the risk of complications. **Methods**: We conducted a prospective study including 28 patients (14 in the control group and 14 in the intervention group) with type I or II diabetes and chronic ulcers of the calf or foot (>4 cm^2^). The control group received standard therapy with debridement, dressings, antibiotics when indicated, and local and systemic ozone therapy. The intervention group was treated with an Integrative Therapeutic Protocol combining ozone therapy, pulsed electromagnetic field therapy (PEMF), colon hydrotherapy with probiotic supplementation, and an anti-inflammatory alkaline diet. Wound healing (reduction in ulcer surface area) was the primary endpoint; secondary endpoints included changes in glycemia and inflammatory biomarkers. **Results**: After 8 weeks, the intervention group achieved 86.2% re-epithelialization versus 58.2% in controls (*p* < 0.01). Significant improvements were also observed in blood glucose level (−38%), HbA1c (−25%), CRP (−26%), and fibrinogen (−28%) relative to baseline, with differences versus controls reaching statistical significance. **Conclusions**: The Integrative Therapeutic Protocol accelerated wound healing and improved glycemic and inflammatory profiles compared with ozone therapy alone. Although an alkaline diet was recommended, adherence and its specific contribution were not objectively monitored; therefore, this component should be interpreted with caution.

## 1. Introduction

### 1.1. Epidemiology of Diabetes Mellitus

Diabetes mellitus (DM) is a chronic, heterogeneous, and multifactorial disease. In addition to genetic predisposition, modifiable risk factors such as urbanization, sedentary lifestyles, hypercaloric diets, obesity, smoking, and dyslipidemia decisively contribute to its global growth [1]. According to the World Health Organization (WHO), an estimated 527 million individuals worldwide were living with diabetes in 2021, and projections indicate a sharp rise to about 853 million by 2050, reflecting a 46% increase within three decades [2].

The global distribution is highly uneven. Data from the International Diabetes Federation (IDF) indicate that in 2025, 11.1% of the adult population (aged 20–79 years)—approximately one in nine people—will live with diabetes, with type 2 DM accounting for 90% of cases. Four out of five patients (81%) reside in low- and middle-income countries, where access to diagnosis and treatment infrastructure is limited. The annual incidence is about 70 million new cases, emphasizing the accelerating global health crisis [2].

In Romania, the epidemiological landscape reflects global trends. In 2022, approximately 1.27 million patients were recorded with DM. The PREDATOR epidemiological study (2012–2014) revealed a prevalence of 11.6%, yet only 6.46% of patients were receiving antihyperglycemic therapy, leaving nearly half of the cases undiagnosed or untreated [3,4]. This diagnostic gap amplifies both clinical and socio-economic burden, as untreated patients frequently progress to complications like retinopathy, nephropathy, neuropathy, diabetic foot syndrome (DFS), and amputations [5,6].

### 1.2. Diabetic Foot Complications

Among DM complications, diabetic foot syndrome (DFS) remains one of the most destructive, representing a significant cause of morbidity, hospitalization, and healthcare costs. According to the WHO definition, DFS is characterized by ulceration and infection of the foot, often with destruction of deep tissues and sometimes bone, depending on the severity of neuropathy and peripheral vascular disease [7]. Epidemiological estimates show that people with diabetes face a 19–34% lifetime risk of developing DFS, compared with approximately 2% in the general population [8].

Within this subgroup, 15–25% of patients develop diabetic foot ulcers (DFUs), which represent the most severe and disabling manifestation of DFS [4,5]. DFUs are associated with slow or incomplete healing, recurrent infection, chronic pain, and impaired mobility, often resulting in a significant reduction in quality of life. Moreover, DFUs are the leading precursor of lower-limb amputations, with about 85% of diabetes-related amputations preceded by a DFU [4].

The natural history of DFUs is determined by systemic metabolic dysfunction and local anatomical factors [6]. Diabetic arteriopathy causes chronic ischemia and tissue hypoxia, impairing vascular supply to the distal lower limb and helping progression toward deep ulcers with necrotic margins, bone exposure, or osteomyelitis [7]. Venous and lymphatic dysfunction worsens edema and local inflammation, while neuropathic sensory loss reduces pain perception, causing patients to neglect early lesions. Furthermore, fragile skin and recurrent microtrauma from footwear or pressure points create constant entry sites for infection [7]. All together, these mechanisms explain why DFUs remain one of the leading causes of non-traumatic amputation worldwide [9].

### 1.3. Pathophysiological Mechanisms of Impaired Healing

The impaired healing of diabetic wounds reflects a multifactorial pathogenesis involving immune dysregulation, oxidative stress, vascular impairment, and microbial colonization.

Chronic inflammation and immune imbalance. Persistent inflammation is a central hallmark. Macrophages are blocked in the M1 pro-inflammatory phenotype and fail to transition to the pro-regenerative M2 phenotype, perpetuating tissue injury. Neutrophils demonstrate impaired chemotaxis and excessive release of neutrophil extracellular traps (NETs). T-cell dysfunction further destabilizes the immune balance. Elevated TNF-α and IL-6 sustain the inflammatory milieu, blocking the progression to the proliferative phase of healing [10].

Angiogenesis failure and fibroblast dysfunction. Deficits of vascular endothelial growth factor (VEGF), epidermal growth factor (EGF), and keratinocyte growth factor (KGF) limit angiogenesis [11]. Fibroblast proliferation, essential for extracellular matrix (ECM) deposition, is inhibited by local acidosis and hypoxia [12].

Oxidative stress and hyperglycemia. Persistent hyperglycemia leads to chronic oxidative stress, altering mitochondrial function in keratinocytes and fibroblasts, impairing migration and collagen synthesis [13,14,15]. Simultaneously, hyperglycemia promotes glycation of structural proteins and disrupts gene expression. Lamers et al. demonstrated that high glucose levels directly impair cell migration through reactive oxygen species (ROS)-mediated signaling [16]. Complementary transcriptomic analyses confirmed that hyperglycemia induces distinct gene expression changes in primary human skin cells, providing molecular evidence for impaired re-epithelialization [17].

Microbial biofilms and ECM degradation. Pathogenic biofilms, commonly formed by *Staphylococcus aureus* and *Pseudomonas aeruginosa*, sustain inflammation and resist eradication. They release toxins that damage tissues, while ECM remodeling is disrupted by an imbalance between matrix metalloproteinases (MMPs) and tissue inhibitors of metalloproteinases (TIMPs). Additionally, impaired macrophage polarization further exacerbates ECM degradation [13,18].

Vascular dysfunction. Advanced glycation end-products (AGEs) promote vascular stiffening and vasoconstriction, while decreased nitric oxide (NO) availability reduces perfusion and aggravates ischemia [13,19].

Collectively, these mechanisms create a “stalled wound” phenotype: chronic, non-healing ulcers marked by persistent inflammation, impaired angiogenesis, oxidative stress, microbial colonization, and defective tissue regeneration [19].

### 1.4. Current Therapies and Limitations

DFS management is multidisciplinary, with the objectives of infection control, wound healing, and recurrence prevention [20,21,22,23]. Standard components include the following:

- Metabolic control: strict regulation of blood glucose level and associated metabolic risk factors.
- Wound care procedures: cleaning, antisepsis, debridement, and appropriate dressings.
- Surgical interventions: ranging from minor drainage to amputations in severe, non-salvageable cases.
- Patient education: focusing on hygiene, protective footwear, and early recognition and reporting of lesions.
- Adjunctive therapies: such as hyperbaric oxygen therapy (HBOT), growth factors, or bioengineered skin substitutes.

Conventional wound dressings (hydrocolloids [23,24], hydrogels [25], silver dressings [26], polyurethane foams [27]) provide partial benefits but fail to accelerate deep wound healing significantly. Recombinant growth factors, including GM-CSF, EGF, KGF, and bFGF, promote cell proliferation and differentiation. Yet, their clinical efficacy is limited by poor diffusion in ischemic tissue, high cost, and adverse reactions [28,29].

Negative pressure wound therapy (NPWT) enhances wound contraction, reduces edema, and promotes granulation tissue formation, but outcomes are diminished in ischemic ulcers, and treatment is costly and lengthy [27]. HBOT aims to improve oxygenation, collagen synthesis, and immune activation, but clinical evidence remains mixed. Risks from comorbidities, restricted chamber availability, and elevated costs further constrain its applicability [30,31,32].

Ozone therapy has gained attention for its multiple benefits. Locally and systemically, it exerts antimicrobial effects via oxidation of microbial membranes and biofilm disruption [33]; downregulates inflammatory mediators such as CRP, IL-6, and TNF-α [34]; enhances antioxidant defenses through catalase, superoxide dismutase, and glutathione peroxidase [35]; stimulates angiogenesis by increasing VEGF, PDGF, and TGF-β [36]; and promotes fibroblast and keratinocyte proliferation [37]. Clinical evidence supports improved healing rates and reduced antibiotic use when ozone therapy is combined with conventional care or NPWT [38,39,40,41].

Pulsed electromagnetic field therapy (PEMF) represents another promising adjunctive therapy. By inducing microcurrents in tissues, PEMF stimulates mitochondrial ATP production, promotes angiogenesis, modulates macrophage polarization toward the regenerative M2 phenotype, and reduces local inflammation [42,43,44,45,46,47,48,49,50]. Devices emit fields at frequencies around 27.12 MHz, which penetrate tissues without causing thermal effects, with exposure durations ranging from minutes to hours [45,46]. Early clinical studies suggest accelerated healing of DFUs, though evidence remains limited [51,52,53]. Current IWGDF guidelines recommend electromagnetic therapies only as adjunctive therapy for ulcers unresponsive to 4–6 weeks of standard therapy [54].

### 1.5. Rationale and Aim

Despite these therapeutic advances, DFUs remain a significant clinical challenge, because conventional methods fail to address the complex relationship of chronic inflammation, oxidative stress, vascular dysfunction, and microbial biofilms.

This study was designed to evaluate an Integrative Therapeutic Protocol that combines established regenerative interventions (ozone therapy and PEMF) with systemic anti-inflammatory and microbiome-targeting measures (colon hydrotherapy with probiotics and an anti-inflammatory dietary regimen).

The primary objective was a reduction in ulcer surface area, reflecting re-epithelialization. Secondary objective included changes in glycemic control (fasting blood glucose, HbA1c), inflammatory biomarkers (CRP, ESR, fibrinogen), anthropometric indices (body weight, BMI), and wound microbiology.

## 2. Materials and Methods

### 2.1. Study Group

The study included 28 patients diagnosed with type I and II DM, with DFS, aged 30–80 years, treated at the Center of Excellence in Ozone Therapy in Bucharest, Romania, with lesions specific to diabetic foot syndrome: calf skin damage, soft tissue infection, and sometimes bone destruction at the toes. This study was conducted under the approval of the Bioethics Committee of “Carol Davila” University of Medicine and Pharmacy in Bucharest, Romania (no. 20157/2025). From the total group of 28 patients, 14 patients constituted the control group (ozone therapy), and 14 patients the intervention group (Integrative Therapeutic Protocol, which combined ozone therapy with three other adjuvant methods: colon hydrotherapy with probiotics, an alkaline anti-inflammatory diet, and low-frequency electromagnetic therapy) (Table 1).

The inclusion criteria were as follows: patients diagnosed with type I or II DM, for more than 5 years, HbA1c < 9%, with varying degrees of neuropathy, who presented with active chronic ulcers of the calf and foot with a surface area > 4 cm^2^. For multiple ulcers, only the largest wound was considered. The presence of soft tissue infection was confirmed clinically and paraclinically.

The Meggitt–Wagner Scale (also known as the Wagner–Meggitt Classification) was used to grade the severity of DFUs [55]:

- Grade 0: Skin intact, but the foot is “at risk” due to existing bony deformities;
- Grade 1: Superficial ulcer, involving only the skin and subcutaneous tissue;
- Grade 2: Deep ulcer with full-thickness extension;
- Grade 3: Deep ulcer with abscess or osteomyelitis;
- Grade 4: Partial gangrene of the foot;
- Grade 5: Extensive gangrene.

The classification in the Meggitt–Wagner Scale was grades 2–4; patients were aged between 30 and 80 years, regardless of gender, occupation, or background. All patients signed informed consent before starting the treatment.

The exclusion criteria included: patients with DM and chronic leg ulcers with absent distal pulse, severe comorbidities (severe kidney disease, cancer, sepsis, corticosteroid therapy, immunosuppressants), contraindications to colon hydrotherapy (bleeding hemorrhoids, anal fissures, colorectal cancer, ulcerative colitis, Crohn’s disease, diverticulitis, pelvic adhesions, pregnancy), contraindications to ozone therapy (glucose-6-phosphate dehydrogenase deficiency, thrombocytopenia, coagulation disorders, massive bleeding, acute inflammation, history of seizures), or contraindications to electromagnetic therapy (high fever, pregnancy, severe cardiovascular disease, pacemaker, metal implants, hearing aids, myasthenia gravis, epilepsy, coagulation disorders). Patients with immobilization, severe psychiatric disorders, refusal to change diet, or insufficient knowledge of the Romanian language were also excluded.

### 2.2. Study Design

Until enrollment in this study, all patients had undergone treatment for the underlying disease with oral antidiabetics (OADs) or insulin, symptomatic therapy, and local interventions—including hydrocolloid dressings, hydrogels, silver, or polyurethane foams—without achieving a positive therapeutic effect.

Prior to allocation, patients were interviewed and underwent a thorough medical evaluation, including assessment of arterial and venous blood flow by Doppler ultrasound. The following parameters were recorded: weight, height, BMI, lifestyle factors (smoking and alcohol consumption), aggravating risk factors (blood glucose level and hemoglobin A1c), bacteriological wound analysis, and markers of chronic systemic inflammation (ESR, FBG, and CRP).

The control group (*n* = 14) received local and systemic ozone therapy twice weekly according to the following protocol: wound debridement and disinfection with ozonized water (at the first session); ozone limb bagging (initial concentration 70 µg/mL, gradually reduced to 40 µg/mL); perilesional infiltrations with oxygen–ozone (5–10 µg/mL); major autohemotherapy with 120–150 mL venous blood ozonated at 25–35 µg/mL; and final dressing with sterile compresses and ozonated oil at the end of each procedure.

The intervention group (*n* = 14) was treated with an Integrative Protocol combining four therapeutic approaches: ozone therapy, colon hydrotherapy, microbiome restoration with probiotics, an anti-inflammatory alkaline diet, and low-frequency electromagnetic therapy.

Antibiotics were prescribed only when wound cultures and antibiogram confirmed bacterial infection, to rule out the possibility that faster wound healing was due to antibiotics alone.

Evaluations were conducted at baseline, at four weeks, and at eight weeks for both groups. Clinical outcomes included wound evolution, time and degree of re-epithelialization, and changes in biological parameters associated with DM and chronic inflammation. Vascular insufficiency was assessed using Doppler ultrasound, and wound severity was evaluated using the Meggitt–Wagner classification.

The Integrative Protocol included the following:

- Initial session: colon hydrotherapy, followed by rectal ozone insufflation (20 µg/mL, one session/week for 3 weeks, then every 2 weeks for 4 weeks), administration of oral probiotics, and adoption of an anti-inflammatory alkaline diet.
- Subsequent sessions (twice weekly):
  - Wound antisepsis and lavage with ozonized water;
  - Wound debridement (performed only at the first session);
  - Antibiotic therapy strictly guided by antibiogram results;
  - Local ozone therapy: limb bagging (70 µg/mL for 3–4 sessions, then gradually reduced to 40 µg/mL) and perilesional infiltrations (5–10 µg/mL);
  - Systemic ozone therapy: major autohemotherapy with 120–150 mL venous blood ozonated at 25–35 µg/mL, provided systolic BP ≤ 160 mmHg;
  - Wound dressing with sterile compresses and ozonated olive oil at the end of each procedure;
  - Pulsed electromagnetic field therapy (20–70 Hz, 10–15 Gauss, 30 min/session, 2 sessions/week), adapted to wound-healing phase.

PEMF/ELF-EMF frequencies were applied according to the ulcer stage and wound-healing phase:

- Phase I—Inflammatory phase: 70–100 Hz, providing anti-inflammatory and analgesic effects by decreasing pro-inflammatory cytokines (TNF-α, IL-1β) and improving microcirculation (~1 week) [56].
- Phase II—Proliferative phase: 12–20 Hz, 10–15 Gauss to stimulate mitochondrial activity and increase ATP production; subsequently, 40–60 Hz were used to enhance angiogenesis, VEGF expression, granulation, collagen deposition, neoangiogenesis, and keratinocyte migration (~1–3 weeks) [57,58].
- Phase III—Tissue maturation and remodeling: 20–30 Hz to promote cellular metabolism and collagen synthesis; toward the final stage, 70 Hz was applied for its anti-inflammatory effect and to improve collagen quality, extracellular matrix remodeling, and re-epithelialization (>3–4 weeks) [59].

### 2.3. Statistical Analysis

All data were introduced in Microsoft Office Excel and IBM SPSS Statistics and IBM SPSS Statistics version 26.0. Descriptive statistics, *t*-tests, and ANOVA were applied where appropriate. A *p*-value < 0.05 was considered statistically significant.

## 3. Results

### 3.1. Participant Flow and Analysis Set

A total of 28 patients with type I or II diabetes mellitus and chronic foot or calf ulcers were enrolled and randomized into two equal groups (*n* = 14 each). All participants completed the study and were analyzed at baseline, 4 weeks, and 8 weeks. No dropouts or protocol deviations occurred.

### 3.2. Baseline Characteristics

At baseline, the two groups were comparable across demographic, clinical, and laboratory parameters, with no statistically significant differences (all *p* > 0.05; Table 2). This confirmed that randomization achieved balanced groups and that subsequent outcome differences could be attributed to the interventions rather than pre-existing disparities.

### 3.3. Primary Outcome: Ulcer Healing

Ulcer area decreased progressively in both groups during the 8-week follow-up, but reductions were significantly greater in the intervention group. At 4 weeks, the mean ulcer area was 5.79 ± 2.19 cm^2^ in the intervention group vs. 7.93 ± 4.14 cm^2^ in controls (*p* = 0.009). By 8 weeks, patients in the intervention group achieved an average wound area of 1.79 ± 1.67 cm^2^ compared with 4.93 ± 3.41 cm^2^ in controls (*p* = 0.005). These values correspond to a mean re-epithelialization of 86.2% in the intervention group vs. 58.2% in controls. Within-group paired analyses further confirmed significant ulcer size reductions from baseline to 8 weeks in both groups (*p* < 0.001 each; Table 3).

### 3.4. Secondary Outcomes

#### 3.4.1. Glycemic Control (Fasting Blood Glucose)

Fasting glucose decreased significantly more in the intervention group than in controls. At 4 weeks, mean values were 162.4 ± 16.8 mg/dL vs. 216.6 ± 37.2 mg/dL (*p* < 0.001). By 8 weeks, patients in the intervention group achieved further reduction to 136.6 ± 9.6 mg/dL compared with 220.4 ± 36.5 mg/dL in controls (*p* < 0.001; Table 4). Detailed inferential statistics are available in Supplementary Tables S3 and S4.

#### 3.4.2. Glycated Hemoglobin (HbA1c)

HbA1c also decreased more markedly in the intervention group. After 4 weeks, mean values were 6.86 ± 1.86% vs. 9.17 ± 1.29% in controls (*p* = 0.001). At 8 weeks, HbA1c levels reached 6.45 ± 0.45% vs. 8.58 ± 2.44% (*p* = 0.004; Table 4).

#### 3.4.3. Inflammatory and Coagulation Markers

CRP and fibrinogen were significantly reduced in the intervention group compared with controls at both follow-ups (all *p* < 0.001).

- CRP: 6.16 ± 0.54 mg/L vs. 7.54 ± 0.69 mg/L (*p* < 0.001) at 4 weeks, and 5.59 ± 0.46 mg/L vs. 7.55 ± 0.62 mg/L (*p* < 0.001) at 8 weeks.
- Fibrinogen: 329.0 ± 26.5 mg/dL vs. 403.9 ± 46.0 mg/dL (*p* < 0.001) at 4 weeks, and 293.1 ± 23.3 mg/dL vs. 406.3 ± 43.6 mg/dL (*p* < 0.001) at 8 weeks.

#### 3.4.4. Weight and BMI

Body weight declined significantly in the intervention group compared with controls (*p* = 0.004 at 4 weeks; *p* = 0.001 at 8 weeks). BMI, however, showed no statistically significant differences between groups. Importantly, the previously reported transcription error was corrected: at 8 weeks, mean BMI values were 27.66 (control) and 26.69 (intervention; Table 4).

### 3.5. Microbiology and Antibiotic Use

At baseline, wound cultures most frequently identified *Staphylococcus aureus*, *Pseudomonas aeruginosa*, and mixed aerobic–anaerobic flora, with a similar distribution across the two groups. Antibiotic therapy was initiated strictly when cultures and antibiograms indicated infection and guided by sensitivity results. The proportion of patients requiring antibiotics did not differ significantly between groups (*p* > 0.05). Detailed microbiological findings and antibiotic use are provided in Table S11 (Supplementary Materials).

### 3.6. Safety and Tolerability

No severe adverse events occurred during the study. Mild, transient erythema was observed in three patients during pulsed electromagnetic field therapy, resolving spontaneously without intervention. No treatment discontinuations or protocol deviations were required. Detailed safety data are available in Table S12 (Supplementary Materials).

**Figure 1:**
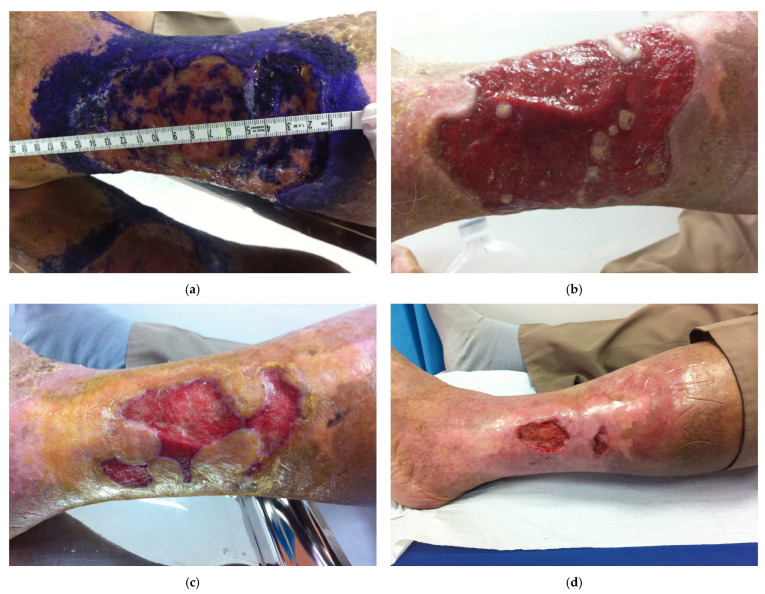
Sequential clinical images showing wound healing progression in a patient treated with the Integrative Therapeutic Protocol: (**a**) baseline ulcer with necrotic tissue; (**b**) after two weeks, granulation tissue formation; (**c**) after four weeks, partial re-epithelialization; (**d**) after eight weeks, near-complete healing.

Representative clinical images documenting wound healing progression in a patient treated with the Integrative Therapeutic Protocol are shown in Figure 1.

## 4. Discussion

### 4.1. Summary of Main Findings

This randomized clinical study demonstrated that the Integrative Therapeutic Protocol—combining ozone therapy, PEMF therapy, colon hydrotherapy with probiotic restoration, and an anti-inflammatory dietary regimen—achieved superior outcomes compared with ozone therapy alone. Patients in the intervention group experienced significantly greater reductions in ulcer area and re-epithelialization, accompanied by improvements in systemic biomarkers, including glycemia, HbA1c, CRP, and fibrinogen. Importantly, these benefits were achieved without significant adverse events, confirming both the feasibility and tolerability of this multimodal approach.

### 4.2. Mechanistic Interpretation

The observed superiority of the Integrative Protocol can be explained by the synergistic interaction of its four components:

Ozone therapy. Consistent with prior reports, ozone exerted multimodal effects by disrupting microbial membranes and biofilms [33], decreasing inflammatory mediators CRP, IL-6, and TNF-α [34], enhancing antioxidant defenses through catalase, superoxide dismutase, and glutathione peroxidase [35], and stimulating angiogenesis via VEGF, PDGF, and TGF-β [36]. Ozone also promoted fibroblast and keratinocyte proliferation, supporting accelerated re-epithelialization [37]. Recent clinical studies confirmed that ozone therapy significantly accelerates DFU healing, reduces oxidative stress, and shortens hospital time [60,61,62]. Molecular analyses highlight ozone’s regulation of redox balance and immunomodulation [63].

PEMF. Pulsed electromagnetic field therapy enhanced mitochondrial ATP production, stimulated angiogenesis, and shifted macrophage polarization from M1 to M2, favoring tissue regeneration [42,43,44,45,46,47,48,49,50]. By reducing oxidative stress and modulating ionic signaling (Ca^2+^, NO), PEMF contributed to local anti-inflammatory effects and collagen synthesis [51,52,53]. These mechanisms complete those of ozone, providing dual stimulation of angiogenesis and ECM remodeling. In addition, recent translational and clinical studies showed that PEMF improves microcirculation and promotes healing of chronic DFUs [64], modulates inflammatory responses [65], and regulates damage-associated molecular pattern release [66], confirming its relevance as a systemic and local bioregulator [67,68,69,70].

Colon hydrotherapy and microbiome modulation. Beyond local wound healing, systemic improvements in glycemic control and inflammatory markers can be attributed to interventions targeting the intestinal ecosystem. Colon hydrotherapy facilitates mechanical removal of residual waste and microbial biofilms, decreasing endotoxin (LPS) production and metabolic endotoxemia associated with obesity and insulin resistance [71,72,73]. Restoration of the microbiome with probiotics promotes recolonization by beneficial taxa such as *Lactobacillus* and *Bifidobacterium*, which support epithelial integrity, produce short-chain fatty acids, and reduce systemic inflammation [74,75,76,77,78,79,80,81]. These effects directly align with the reductions in CRP and fibrinogen observed in our study.

Anti-inflammatory alkaline diet. Dietary modulation acted synergistically by reducing systemic inflammation and oxidative stress while supporting collagen synthesis and tissue regeneration. A lower Dietary Inflammatory Index (DII) score is associated with decreased CRP, IL-6, and TNF-α [82,83], while reduced dietary acid load (low PRAL) improves mineral balance and supports angiogenesis [83]. Key nutrients—such as vitamin C, magnesium, and amino acids (proline, glycine, lysine)—are critical for collagen formation [84,85,86]. Antioxidants (vitamins C and E, carotenoids, polyphenols) mitigate oxidative stress, while low glycemic load foods prevent hyperglycemia-driven immune dysregulation [87,88,89,90,91,92,93,94]. Collectively, this dietary intervention created a systemic environment favoring re-epithelialization and metabolic stabilization.

### 4.3. Comparison with Existing Literature

Our findings are consistent with randomized trials demonstrating accelerated DFU healing with ozone therapy, where complete healing was achieved in 81% of patients versus 44% in controls, and with large series showing shorter healing times and reduced antibiotic use [40,41,95]. These findings are also in line with recent experimental and clinical studies that evaluated ozone therapy and PEMF in DFU and wound healing contexts [55,56,57,58,59]. The observed systemic anti-inflammatory effects of ozone corroborate previous evidence of reduced CRP and improved peripheral circulation [39]. More recently, retrospective and clinical studies confirmed the efficacy of ozone as an adjuvant therapy in DFUs, highlighting reductions in oxidative stress, faster wound closure, and lower recurrence rates [60,61]. Meta-analyses further support ozone as a valuable adjunct in diabetic wound care [62].

Similarly, our results extend prior experimental data on PEMF, where collagen synthesis, angiogenesis, and macrophage modulation were reported [44,48,49,50], by demonstrating clinical efficacy in human subjects. Clinical pilot studies showed that PEMF enhances microcirculation and promotes ulcer healing [64]. Systematic reviews confirmed its therapeutic relevance in tissue repair and osteoarticular disorders [68], while translational studies underlined PEMF’s ability to regulate inflammatory pathways [65] and DAMP signaling [66]. These data corroborate our results and suggest that PEMF acts as a systemic bioregulator with potential applications beyond DFU [67,69,70].

The incorporation of colon hydrotherapy and microbiome restoration represents a new dimension. While prior studies have focused on metabolic endotoxemia and gut dysbiosis in diabetes [74,75,76], few have linked these processes to wound healing. By reducing LPS load and restoring epithelial barrier function, our protocol directly addressed systemic inflammatory drivers of impaired regeneration.

The dietary component aligns with epidemiological data showing that low-inflammatory and low-acid load diets improve metabolic control and support tissue healing [82,83,96,97,98]. Nutritional interventions enriched in antioxidants and omega-3 fatty acids were shown to reduce systemic inflammation, enhance endothelial function, and accelerate wound healing. This further supports the observed improvements in our trial.

Thus, our study is among the first to demonstrate the combined clinical benefits of local regenerative (ozone, PEMF) and systemic anti-inflammatory (colon hydrotherapy, microbiome restoration, diet) therapies in DFU, providing a precision integrative approach consistent with recent translational evidence [61,63,64].

### 4.4. Clinical Implications

The Integrative Protocol addresses the multifactorial pathogenesis of DFUs, simultaneously targeting local ulcer microenvironments and systemic drivers of inflammation and metabolic dysfunction. Clinically, this integrative model may achieve the following:

- Reduce healing time and accelerate re-epithelialization.
- Lower systemic inflammation and improve metabolic control.
- Reduce antibiotic use and potentially decrease amputation risk.

This paradigm reflects a precision-integrative medicine approach, combining regenerative, metabolic, and microbiome-targeted interventions for complex diabetic complications.

## 5. Limitations

This study has several limitations. The small sample size (*n* = 28) and the relatively short follow-up period of eight weeks limit the generalizability of the findings and the capacity to assess long-term outcomes. Furthermore, the open-label design may have introduced bias, and adherence to dietary modification and probiotic supplementation relied on self-reported measures, which are subject to variability and potential inaccuracy. Colon hydrotherapy remains a controversial intervention with limited large-scale evidence; no molecular or microbiome analyses have been performed to clarify the mechanisms of wound healing.

## 6. Suggestions for Research

Larger, multicenter randomized controlled trials with extended follow-up are needed to confirm the efficacy and durability of the Integrative Therapeutic Protocol. Comparative studies should isolate the specific contributions of each component (ozone therapy, PEMF, colon hydrotherapy, probiotics, and diet) and investigate the potential synergistic effects. Incorporating molecular and microbiome profiles could elucidate the biological mechanisms underlying re-epithelialization and the reduction in systemic inflammation. Evaluating patient-reported outcomes, cost-effectiveness, and adherence would provide valuable insights for the clinical applicability of these findings. Finally, integrating precision medicine approaches, such as biomarker- or genetics-based stratification, may optimize patient selection and maximize therapeutic benefit.

## 7. Conclusions

This study evaluated the therapeutic effect of simultaneously applying multiple synergistic interventions on the re-epithelialization of diabetic foot wounds. Our findings demonstrated that combining ozone therapy, low-frequency electromagnetic therapy, detoxification, and dietary modification enhanced wound healing in diabetic patients with chronic ulcers. Both ozone therapy and low-frequency electromagnetic therapy had been independently shown to facilitate tissue repair; however, their combined application accelerates wound healing, particularly when accompanied by detoxification and dietary modification.

## Figures and Tables

**Table 1:** The groups of patients were divided according to the interventions.

Group	Intervention	No. of Patients
G1-Control	Standard treatment (debridement, dressings, antibiotics when indicated by wound culture and antibiogram) + local and general ozone therapy	14
G2-Protocol	Standard treatment + local and general ozone therapy + probiotic hydrocolonotherapy + alkaline diet + PEMF (20–70 Hz)	14

**Table 2:** Baseline characteristics of study groups (mean ± SD; *n* = 14 each).

Parameter	Control Group (*n* = 14)	Intervention Group (*n* = 14)	*p*-Value
Age (years)	61.4 ± 8.2	60.7 ± 7.9	0.78
Male/Female	8/6	9/5	0.68
Diabetes type I/II	4/10	5/9	0.71
Duration of diabetes (years)	12.3 ± 6.1	13.0 ± 5.8	0.65
Ulcer area (cm^2^)	11.79 ± 5.41	13.00 ± 6.04	0.58
Fasting blood glucose (mg/dL)	191.1 ± 25.4	183.8 ± 21.6	0.42
HbA1c (%)	7.67 ± 2.09	8.02 ± 0.75	0.56
Weight (kg)	84.4 ± 4.0	83.8 ± 4.0	0.67
BMI (kg/m^2^)	25.9 ± 10.1	30.0 ± 1.6	0.15
CRP (mg/L)	7.49 ± 0.64	7.05 ± 0.53	0.06
Fibrinogen (mg/dL)	399.9 ± 48.1	369.5 ± 32.2	0.06

Notes: Detailed inferential statistics (Levene’s test, *t*-test, 95% CI) are available in the Supplementary Information (Table S1).

**Table 3:** Ulcer area (cm^2^) at baseline, 4 weeks, and 8 weeks (mean ± SD). ^†^ *p* < 0.05; ^‡^ *p* < 0.01 vs. control at the same timepoint.

Timepoint	Control	Intervention	*p*-Value
Baseline	11.79 ± 5.41	13.00 ± 6.04	0.58
4 weeks	7.93 ± 4.14	5.79 ± 2.19 ^†^	0.009
8 weeks	4.93 ± 3.41	1.79 ± 1.67 ^‡^	0.005

Notes: Between-group tests are shown above; detailed test statistics and confidence intervals are provided in Supplementary Tables S2–S4.

**Table 4:** Secondary outcomes at 4 and 8 weeks (mean ± SD). ^‡^ *p* < 0.01; ^§^ *p* < 0.001 vs. control at the same timepoint.

Parameter	Control 4w	Intervention 4w	*p*-Value	Control 8w	Intervention 8w	*p*-Value
Fasting glucose (mg/dL)	216.6 ± 37.2	162.4 ± 16.8 ^§^	<0.001	220.4 ± 36.5	136.6 ± 9.6 ^§^	<0.001
HbA1c (%)	9.17 ± 1.29	6.86 ± 1.86 ^‡^	0.001	8.58 ± 2.44	6.45 ± 0.45 ^‡^	0.004
CRP (mg/L)	7.54 ± 0.69	6.16 ± 0.54 ^§^	<0.001	7.55 ± 0.62	5.59 ± 0.46 ^§^	<0.001
Fibrinogen (mg/dL)	403.9 ± 46.0	329.0 ± 26.5 ^§^	<0.001	406.3 ± 43.6	293.1 ± 23.3 ^§^	<0.001
Weight (kg)	85.3 ± 4.0	82.0 ± 3.8 ^‡^	0.004	85.3 ± 3.7	79.7 ± 3.7 ^§^	0.001
BMI (kg/m^2^)	27.85 ± 7.69	29.38 ± 1.56	0.475	27.66 ± 7.6	26.69 ± 7.0	0.729

Notes: Full inferential statistics (Levene’s test, t values, 95% CI) are reported in Supplementary Tables S5–S14.

## Data Availability

The original contributions presented in this study are included in the article and Supplementary Material. Further inquiries can be directed to the corresponding authors.

## Supplementary Materials

The following supporting information can be downloaded at: https://www.mdpi.com/article/10.3390/bioengineering12101053/s1, Tables S1–S14: Independent Samples Test.

## Abbreviations

The following abbreviations are used in this manuscript:

DM	diabetes mellitus
WHO	World Health Organization
IDF	International Diabetes Federation
DFS	diabetic foot syndrome
DFU	diabetic foot ulcers
MMPs	matrix metalloproteinases
AGEs	advanced glycation end-products
NO	nitric oxide
GM-CSF	granulocyte–macrophage colony-stimulating factor
EGF	epidermal growth factor
KGF-2/FGF-10	keratinocyte growth factor-2/paracrine growth factor 10
bFGF/FGF-2	basic fibroblast growth factor
NPWT	negative pressure wound therapy
HBOT	hyperbaric oxygen therapy
PEMF	pulsed electromagnetic field
VEGF	vascular endothelial growth factor
FBG	fibrinogen
ESR	erythrocyte sedimentation rate
CRP	C-reactive protein
BMI	body mass index
