# Supplementary material for: Promoting Re-Epithelialization in Diabetic Foot Wounds Using Integrative Therapeutic Approaches
Source: Bioengineering (Basel). 2025 Sep 29;12(10):1053. doi: 10.3390/bioengineering12101053 (PMC12561719; doi:10.3390/bioengineering12101053)
Supplement: Supplementary file 1 [file bioengineering-12-01053-s001.zip › bioengineering-3877467-supplementary.pdf]

Supplementary Materials

Table S1. Independent Samples Test.

		Levene's Test for Equality of Variances		t-test for Equality of Means						
		F	Sig.	t	df	Sig. (2-tailed)	Mean Difference	Std. Error Difference	95% Confidence Interval of the Difference	
									Lower	Upper
Wound (cm)	Equal variances assumed	5.829	.023	1.712	26	.009	2.143	1.252	-.431	4.716
	Equal variances not assumed			1.712	19.745	.010	2.143	1.252	-.471	4.757

Table S2. Independent Samples Test.

		Levene's Test for Equality of Variances		t-test for Equality of Means						
		F	Sig.	t	df	Sig. (2-tailed)	Mean Difference	Std. Error Difference	95% Confidence Interval of the Difference	
									Lower	Upper
Wound (cm)	Equal variances assumed	7.545	.011	3.098	26	.005	3.143	1.014	1.058	5.228
	Equal variances not assumed			3.098	18.920	.006	3.143	1.014	1.019	5.267

Table S3. Independent Samples Test.

		Levene's Test for Equality of Variances		t-test for Equality of Means						
		F	Sig.	t	df	Sig. (2-tailed)	Mean Difference	Std. Error Difference	95% Confidence Interval of the Difference	
									Lower	Upper
Blood glucose (mg/dL)	Equal variances assumed	9.662	.005	4.963	26	.000	54.21429	10.92297	31.76180	76.66677
	Equal variances not assumed			4.963	18.105	.000	54.21429	10.92297	31.27549	77.15308

Table S4. Independent Samples Test.

		Levene's Test for Equality of Variances		t-test for Equality of Means						
		F	Sig.	t	df	Sig. (2-tailed)	Mean Difference	Std. Error Difference	95% Confidence Interval of the Difference	
									Lower	Upper
Blood glucose (mg/dL)	Equal variances assumed	18.157	.000	8.299	26	.000	83.71429	10.08765	62.97883	104.44974
	Equal variances not assumed			8.299	14.776	.000	83.71429	10.08765	62.18451	105.24406

Table S5. Independent Samples Test.

		Levene's Test for Equality of Variances		t-test for Equality of Means						
		**F**	**Sig.**	**t**	**df**	**Sig. (2-tailed)**	**Mean Difference**	**Std. Error Difference**	**95% Confidence Interval of the Difference**	
									**Lower**	**Upper**
HbA1c (%)	Equal variances assumed	.060	.808	3.797	26	.001	2.30	0.60	1.05	3.55
	Equal variances not assumed			3.797	23.170	.001	2.30	0.60	1.05	3.56

Table S6. Independent Samples Test.

		Levene's Test for Equality of Variances		t-test for Equality of Means						
		F	Sig.	t	df	Sig. (2-tailed)	Mean Difference	Std. Error Difference	95% Confidence Interval of the Difference	
									Lower	Upper
HbA1c (%)	Equal variances assumed	4.865	.036	3.200	26	.004	2.12	0.66	0.76	3.49
	Equal variances not assumed			3.200	13.904	.006	2.12	0.66	0.70	3.55

Table S7. Independent Samples Test

		Levene's Test for Equality of Variances		t-test for Equality of Means						
		F	Sig.	t	df	Sig. (2-tailed)	Mean Difference	Std. Error Difference	95% Confidence Interval of the Difference	
									Lower	Upper
Weight (kg)	Equal variances assumed	.000	1.000	2.236	26	.004	3.28571	1.46920	.26573	6.30570
	Equal variances not assumed			2.236	25.896	.004	3.28571	1.46920	.26514	6.30629

Table S8. Independent Samples Test.

		Levene's Test for Equality of Variances		t-test for Equality of Means						
		F	Sig.	t	df	Sig. (2-tailed)	Mean Difference	Std. Error Difference	95% Confidence Interval of the Difference	
									Lower	Upper
Weight (kg)	Equal variances assumed	.007	.933	3.974	26	.001	5.57143	1.40195	2.68968	8.45318
	Equal variances not assumed			3.974	25.997	.001	5.57143	1.40195	2.68966	8.45320

Table S9. Independent Samples Test.

		Levene's Test for Equality of Variances		t-test for Equality of Means						
		F	Sig.	t	df	Sig. (2-tailed)	Mean Difference	Std. Error Difference	95% Confidence Interval of the Difference	
									Lower	Upper
BMI (kg/m²)	Equal variances assumed	2.389	.134	-.725	26	.475	-1.52	2.09	-5.83	2.79
	Equal variances not assumed			-.725	14.075	.480	-1.52	2.09	-6.02	2.97

Table S10. Independent Samples Test.

		Levene's Test for Equality of Variances		t-test for Equality of Means						
		F	Sig.	t	df	Sig. (2-tailed)	Mean Difference	Std. Error Difference	95% Confidence Interval of the Difference	
									Lower	Upper
BMI (kg/m²)	Equal variances assumed	.061	.806	.351	26	.729	96.85714	276.17997	-470.83892	664.55321
	Equal variances not assumed			.351	25.854	.729	96.85714	276.17997	-470.99511	664.70940

Table S11. Independent Samples Test.

		Levene's Test for Equality of Variances		t-test for Equality of Means						
		F	Sig.	t	df	Sig. (2-tailed)	Mean Difference	Std. Error Difference	95% Confidence Interval of the Difference	
									Lower	Upper
CRP (mg/L)	Equal variances assumed	1.479	.235	5.903	26	.000	1.37857	.23354	.89852	1.85862
	Equal variances not assumed			5.903	24.589	.000	1.37857	.23354	.89718	1.85996

Table S12. Independent Samples Test.

		Levene's Test for Equality of Variances		t-test for Equality of Means						
		F	Sig.	t	df	Sig. (2-tailed)	Mean Difference	Std. Error Difference	95% Confidence Interval of the Difference	
									Lower	Upper
CRP (mg/L)	Equal variances assumed	.390	.538	9.530	26	.000	1.96429	.20612	1.54061	2.38797
	Equal variances not assumed			9.530	24.154	.000	1.96429	.20612	1.53902	2.38955

Table S13. Independent Samples Test.

		Levene's Test for Equality of Variances		t-test for Equality of Means						
		F	Sig.	t	df	Sig. (2-tailed)	Mean Difference	Std. Error Difference	95% Confidence Interval of the Difference	
									Lower	Upper
FBG (mg/dL)	Equal variances assumed	11.336	.002	5.280	26	.000	74.85714	14.17817	45.71350	104.00078
	Equal variances not assumed			5.280	20.754	.000	74.85714	14.17817	45.35075	104.36354

Table 14. Independent Samples Test.

		Levene's Test for Equality of Variances		t-test for Equality of Means						
		F	Sig.	t	df	Sig. (2-tailed)	Mean Difference	Std. Error Difference	95% Confidence Interval of the Difference	
									Lower	Upper
FBG (mg/dL)	Equal variances assumed	14.362	.001	8.572	26	.000	113.14286	13.19935	86.01120	140.27452
	Equal variances not assumed			8.572	19.846	.000	113.14286	13.19935	85.59578	140.68993
